# Relationship between quality attributes of backslopped fermented gari and the sensory and instrumental texture profile of the cooked dough (*eba*)

**DOI:** 10.1111/jfpp.16115

**Published:** 2021-11-14

**Authors:** Wasiu Awoyale, Hakeem Oyedele, Ayodele A. Adenitan, Michael Adesokan, Emmanuel O. Alamu, Busie Maziya‐Dixon

**Affiliations:** ^1^ International Institute of Tropical Agriculture Ibadan Nigeria; ^2^ Department of Food Science & Technology Kwara State University Malete Ilorin Nigeria; ^3^ International Institute of Tropical Agriculture (IITA) Lusaka Zambia

## Abstract

Knowledge is scarce on the drivers of textural characteristics of cooked dough prepared from gari (*eba*). To address this need, quality attributes of backslopped fermented gari (BFG) were correlated with the sensory texture profile analysis (STPA) and instrumental texture profile analysis (ITPA) of the eba produced from six cassava varieties. The results showed that a significant and negative correlation exists between the STPA mouldability of the eba and the sugar (*p* < .05, *r* = −0.83) and amylose (*p* < .05, *r* = −0.86) contents of the BFG. The STPA stretchability of the eba had a significant negative correlation with the bulk density (*p* < .05, *r* = −0.83) and a significant positive correlation with the setback viscosity (*p* < .01, *r* = 0.92) of the BFG. A significant and negative correlation exists between the STPA stickiness of the eba and the amylose (*p* < .05, *r* = −0.84) contents of the BFG. The ITPA adhesiveness of the eba was significant and negatively correlated with the solubility index (*p* < .05, *r* = −0.89) and the ash content (*p* < .05, *r* = −0.87) of the BFG. The correlation between the ITPA stretchability of the eba and the peak (*p* < .05, *r* = 0.83) and breakdown (*p* < .05, *r* = 0.88) viscosities of the BFG was significant and positive, while a significant negative correlation (*p* < .05, *r* = −0.83) exist between the ITPA stretchability of the eba and the starch content of the BFG. The information provided in this study may assist the breeders in developing varieties with good textural attributes for eba.

**Practical applications:**

This study depicts the relationship between the quality attributes of backslopped fermented gari (BFG) and the sensory and instrumental texture profile of the cooked dough (eba). Both the sensory and the instrumental texture attributes of the eba have correlation with the quality attributes of the BFG. Apart from providing information on the drivers of the textural characteristics of eba, this study may also assist the breeders in developing varieties with good textural attributes for eba.

## 1 INTRODUCTION

The texture of starchy foods is the sensory and functional manifestation of the rheological and biophysical attributes of foods detected through the human feelings and kinesthetic qualities (Civille & Ofteda, 2012; Szczesniak, 2002). The texture of cooked root and tuber crops is often cited as a primary determinant of the consumer acceptability of improved and local cassava varieties. Up till now, cassava breeders have had limited information on the genetic variation related to consumer‐preferred textural characteristics (Goddard et al., 2015).

Breeding for well‐defined attributes, such as percentage yield, resistance to disease, root/tuber shape and size, and nutritional composition, relies on more‐or‐less accepted quantitative metrics and often on well‐understood genetic processes. Consequently, such breeding efforts can be practiced relatively cost‐effectively, with results observed in early breeding generations (Goddard et al., 2015). However, breeding for poorly understood traits that require a subjective taste panel is an expensive project, subject to high variance and requiring a lengthier breeding process (to reduce the numbers of selected lines for taste and sensory evaluation), significantly delaying the development and release of new varieties and their adoption (Van Oirschot et al., 2003). If breeders had laboratory assessment for texture‐associated traits, whether biochemical, biophysical, or genetic, the expectation is that they could develop more quickly and effectively new varieties with characteristics desired by consumers, including consistent or improved palatability (Goddard et al., 2015).

Farmers select cassava varieties to meet their income, food security, culinary, and agronomic needs. They are also selected based on the necessity to maintain their traditional identity while nurturing both the high‐yielding varieties introduced by researchers and the high‐yielding local varieties (Awoyale et al., 2020). Cassava root has been processed into different value‐added products like *lafun*, *fufu,* and *gari*/*eba* (Awoyale et al., 2020). Gari is a roasted spontaneously fermented granule, consumed raw, soaked in cold water, or reconstituted in hot water into eba, although gari can also be produced through the backslopped fermentation method (Awoyale, et al., 2021; Awoyale et al., 2021a). Backslopped fermentation is the use of freshly prepared cassava mash and pre‐fermented cassava mash for gari production. Eba is a dough prepared by stirring gari in boiled water to a preferred texture (Awoyale et al., 2020). Knowledge of the textural attributes of eba produced from different varieties of cassava will reduce the challenges of balancing the requirements of farmers with those of processors and end‐users in terms of their preferred quality traits.

This study aimed to assess the relationship between the functional and pasting properties and the chemical composition of backslopped fermented gari (BFG) with the sensory and instrumental texture profile of the cooked dough (*eba*).

## 2 MATERIALS AND METHODS

### 2.1 Materials

Six cassava varieties were harvested from the IITA NextGen project farm at Ikenne, Ogun State, Nigeria. About 20 kg of each variety was used to produce the BFG.

### 2.2 Production of backslopped fermented gari (BFG)

About 5 kg of each cassava variety was processed into a mash after peeling, washing, and grating. The cassava mash was pre‐fermented for about 96 hr in a black‐colored plastic container (backslopped cassava mash‐BCM). *Lactobacillus fermentum* was identified in the BCM (Awoyale et al., 2021a). About 15 kg of freshly harvested, peeled, washed, and grated roots (fresh cassava mash‐FCM) was adequately mixed with BCM at a ratio of 87.02%FCM:20%BCM. The blended mash was then bagged, dewatered, granulated, sifted, and roasted manually in the laboratory using a stainless‐steel roasting pan mounted on an electric cooker. An infrared thermometer was used to monitor the roasting temperature and maintained as much as possible at between 68 and 70°C for about 20 min. The roasted gari was then cooled and packaged for further study (Awoyale et al., 2021a).

### 2.3 Determination of functional properties

#### 2.3.1 Bulk density

The gari samples (10 g) were measured into a 50 ml graduated measuring cylinder and gently tapped on the bench 10 times to achieve a constant height of the contents. The sample volume was recorded and expressed as grams per milliliter (Ashraf et al., 2012).

#### 2.3.2 Swelling power and solubility index

The gari samples swelling power (SWP) and solubility index (SI) was determined using the method described by Afoakwa et al. (2012). About 2.5% aqueous starch dispersion of the gari samples was placed in centrifuge tubes, covered to prevent spilling, and heated in a shaker water bath (Precision Scientific, Model 25: Chicago, USA) for 30 min at a temperature of about 85°C. The tubes were allowed to cool to ambient temperature and, following centrifugated heating (Thelco GLC‐ 1, 60,647: Chicago, USA) for 15 min at 3,000 rpm. The paste was, removed from the supernatant and weighed. A hot air oven (Mmert GmbH + Co.KG: D‐91126, Germany) was used to evaporate the liquid above the sediment at 105°C, and the residue was weighed. All determinations were done in duplicates. The SWP and SI were calculated as follows:

$$\text{SWP =}\;\frac{\text{Wt}\;\text{of precipitated}\;\text{paste}}{\text{Wt of sample}}‐\text{wt}\;\text{of residue in supernatant}\times 100$$

$$\text{SI =}\;\frac{\text{Wt}\;\text{of reisdue}\;\text{in supernatant}}{\text{Wt}\;\text{of sample}}\times 100$$

#### 2.3.3 Water absorption capacity

The gari sample (1 g) was weighed into a clean pre‐weighed and dried centrifuge tube and mixed adequately with distilled water (10 ml) by vortexing. The suspension was allowed to stand for 30 min and centrifuged (Thelco GLC‐ 1, 60,647: Chicago, USA) at 3,500 rpm for 30 min. After centrifuging, the supernatant was decanted, and the tube with the sediment was weighed after removing the adhering drops of water. The weight of water (g) retained in the sample was reported as the water absorption capacity (WAC) (Oyeyinka et al., 2013).

#### 2.3.4 Dispersibility

A sample of 10 g was dispersed in distilled water in a 100 ml measuring cylinder, and distilled water was added up to the 50 ml mark. The mixture was stirred vigorously and allowed to settle for 3 hr. The volume of settled particles was noted, and the percentage was calculated (Asaam et al., 2018).

$$\text{Dispersibility}\left(\%\right)=\frac{\left(50‐\text{volume}\;\text{of}\;\text{the settled particles}\right)}{50}\times 100$$

### 2.4 Determination of pasting properties

The pasting properties of the gari sample were measured using a Rapid Visco Analyzer (Model RVA 4500, Perten Instrument, and Australia) equipped with a 1000 cmg sensitivity cartridge. The gari sample (3.5 g) was weighed into a dried empty canister, and 25 ml of distilled water was added. The mixture was thoroughly stirred, and the canister was fitted into the RVA as recommended. The slurry was heated from 50 to 95°C at a rate of 1.5°C/min, held at this temperature for 15 min, then cooled to 50°C. Viscosity profile indices recorded from the pasting profile with the aid of Thermocline for Windows Software connected to a computer were peak viscosity, trough, breakdown, final viscosity setback, peak time, and pasting temperature (Donaldben et al., 2020).

### 2.5 Determination of chemical composition

#### 2.5.1 Starch and sugar contents

The method described by Onitilo et al. (2007) was used for the starch and sugar contents determination. This included weighing 0.02 g of the gari sample into a centrifuge tube with about 1 ml of ethanol, 2 ml of distilled water, and hot ethanol (10 ml). The mixture was vortexed and centrifuged at 2000 rpm for 10 min. The supernatant was poured out and used for determining sugar content. The starch content was then estimated using the sediment hydrolyzed with perchloric acid. Phenol and sulfuric acid reagents were used for color development; glucose standards were used to estimate sugar. The absorbance was read with a spectrophotometer (Genesys 10S UV‐VIS, China) at 490 nm:

$$\%\text{Sugar =}\;\frac{\left(A‐1\right)\times D.F\times V\times 100}{B\times W\times 10^{6}}$$

$$\%\text{Starch}\frac{\left(A‐1\right)\times D.F\times V\times 100\times 0.9}{B\times W\times 10^{6}}$$

where *A* = Absorbance of sample; *I* = Intercept of sample; D.F = Dilution factor (depends on aliquot taken for assay); *V* = volume; *B* = Slope of the standard curve; *W* = Weight of the sample.

#### 2.5.2 Amylose content

The amylose content of samples was determined according to the method described by Mohana et al. (2007). Exactly 0.1 g of gari sample was weighed into a test tube. To this, 1 ml of 95% ethanol and 9 ml of 1 N NaOH were carefully added and vortexed with the test tube mouth covered. The samples were heated for 10 min in a boiling water bath to gelatinize the starch and cooled to room temperature. A 10‐times dilution of the extract was made by taking 1 ml and making this up to 10 ml with 9 ml of water. From the diluents, an aliquot of 0.5 ml was taken for analysis. To this, 0.1 ml of the acetic acid solution and 0.2 ml of iodine solution were added. About 9.2 ml of distilled water was used to make up to 10 ml. The test mixture was left for 20 min. for color development, after which it was vortexed, and the absorbance was read at 620 nm:

$$\%\text{Amylose}\;\text{of}\;\text{sample =}\;\frac{\%\text{Amylose}\;\text{of}\;\text{standard}\times \text{Absorbance}\;\text{of sample}}{\text{Absorbance}\;\text{of standard}}.$$

#### 2.5.3 Ash content

A well‐labeled ash‐crucible containing the weighed sample (3 g) was placed inside the furnace (VULCANTM furnace model 3‐1750) operated at 600°C for 5 hr to burn off moisture and all organic constituents. The ash content was recorded as the weight of the residue after incineration (AOAC, 2000).

#### 2.5.4 pH‐value

The pH of the gari samples was determined using the method of AOAC (2000). The gari sample (10 g) was put in a 100 ml beaker, and 100 ml of distilled water was added. The pH was analyzed using a standardized pH meter (Mettler Toledo GmbH; 8606 Greifensee, Switzerland). Triplicate values were obtained, and the mean was taken as a pH value.

#### 2.5.5 Cyanogenic potential

Thirty grams (30 g) of the gari samples were mixed with about 250 ml of 0.1 M orthophosphoric acid, the mixture was centrifugated, and the supernatant was obtained. The supernatant (0.1 ml) was treated with linamarin standard to get the total cyanogenic potential (CNP). Another analysis was done using 0.1 ml of 0.1M phosphate buffer (pH 6.0) to give the non‐glucosidic CNP. A third analysis was run with the extract (0.6 ml) added to 3.4 ml of McIlvaine buffer (pH 4.5). This was properly mixed, with the addition of 0.2 ml of 0.5% chloramine T and 0.8 ml of a color reagent to give the free cyanogen (Essers et al., 1993). A plot of absorbance value (*y*‐axis) against standard concentration (x‐axis) was plotted to obtain a standard curve: linamarin = 125 ml/(sample wt × 0.01093); Non‐glucosidic cyanogen = 125 ml/(sample wt × 0.03176); free cyanide = 125 ml/(sample wt × 0.04151).

#### 2.5.6 Protein content

The crude protein was determined by a Kjeldahl method using KjeltecTM model 2300 protein analyzer, as described in the Foss Analytical Manual, AB. (2003). About 0.2 g of sample was digested at 420°C for 1 hr to liberate the organically bound nitrogens in the form of ammonium sulphate. The ammonia in the digest (ammonium sulphate) was then distilled off into a boric acid receiver solution and then titrated with standard hydrochloric acid. A conversion factor of 6.25 was used to convert from total nitrogen to percentage crude protein (displayed on the screen of the protein analyzer).

#### 2.5.7 Fat content

Fat was determined using AOAC (2000) method. Crude fat was extracted from 3 g of the sample with hexane using a fat extractor (Soxtec System HT‐2 fat extractor), and the solvent was evaporated off to get the fat. The difference between the initial and final weight of the extraction cup was recorded as the crude fat content:

$$\%\text{Fat}\;\text{content =}\;\left(\frac{\text{Wt}.\text{of}\;\text{flask + fat}‐\text{Wt}.\;\text{of the sample after drying}}{\text{Wt}.\;\text{of the sample before drying}}\times 100\right).$$

### 2.6 Preparation of eba for sensory and instrumental texture profile analysis

Eba was prepared from gari produced from different cassava varieties using the method reported by Udoro et al. (2014). The eba was prepared by adding gari (100 g) to 195 ml of boiled water and continuously stirred with a wooden stirrer to form a smooth, thick paste. Sensory and instrumental texture profile analyses were carried out simultaneously. The sensory texture profile analysis (STPA) was done based on parameters, such as mouldability, stretchability, stickiness, and hardness, using 15 trained panelists from staff and graduate students at the International Institute of Tropical Agriculture, Ibadan, who consumed eba regularly. The sensory evaluation of this study followed the tenets of the Declaration of Helsinki promulgated in 1964 and approved by the International Institute of Tropical Agriculture review committee on ethics. A verbal agreement was also obtained from the participants before the sensory evaluation.

The instrumental texture profile analysis (ITPA) was done using a Texture analyzer (TA‐XTPlus‐Stable Microsystems) equipped with a 50 kg load cell. The parameters derived from the texture analyzers used are the hardness, adhesiveness, resilience, cohesiveness, springiness/stretchability, and gumminess of the eba. It is imperative to add that the eba samples were placed inside a closed container to maintain the temperature (28–30°C).

### 2.7 Statistical analysis

The analysis of variance (ANOVA), separation of means and the Pearson correlation of the generated data were analyzed using the Statistical Package for Social Scientist (SPSS version 21). All the functional and pasting properties, and the chemical composition were done in duplicates. The STPA was done in duplicates using 15 panelists, and the ITPA was done six times for each sample.

## 3 RESULTS AND DISCUSSION

### 3.1 **Functional properties of** BFG **from different cassava varieties**

The functional properties of foods, how they behave during preparation for consumption (Awoyale et al., 2015) are known to affect their end‐use. The functional properties of the BFG mean values is WAC 577%, SWP 14%, SI 15%, bulk density (BD) 58%, and dispersibility 32% (Table 1). Significant differences (*p* < .05) exist in the SI, BD, and dispersibility of the BFG produced from different cassava varieties, but no significant difference (*p* > .05) was observed in WAC and SWP of the BFG samples (Table 1).

**TABLE 1:** Functional properties of backslopped fermented gari produced from different cassava varieties

Varieties	Water absorption capacity (%)	Swelling power (%)	Solubility index (%)	Bulk density (%)	Dispersibility (%)
IITA‐TMSIBA000070	548.74 ± 47.88ab	16.11 ± 0.04a	23.00 ± 4.96a	65.61 ± 1.52a	36.00 ± 1.41b
NR292D	627.14 ± 15.81a	14.82 ± 0.53a	15.39 ± 0.83bc	66.68 ± 0.01a	43.50 ± 0.71a
TMEB419	520.55 ± 39.84b	8.31 ± 10.02a	9.76 ± 3.40c	60.62 ± 0.00b	32.00 ± 0.00bc
NR130022	609.36 ± 12.83ab	16.19 ± 0.30a	17.53 ± 1.55ab	50.67 ± 0.90d	28.50 ± 4.95c
TMS13F1343P0044	572.23 ± 55.12ab	13.40 ± 1.09a	12.76 ± 1.33bc	53.35 ± 1.01c	22.00 ± 0.00d
TMSF1153P001	584.53 ± 24.04ab	12.77 ± 0.30a	11.25 ± 1.07bc	49.42 ± 0.87d	28.50 ± 2.12c
Mean	577.09	13.6	14.95	57.72	31.75
*p* level	NS	NS	*	***	**

Note

Means with different letters within the same column are significantly different at *p* < .05.

Abbreviation: NS, not significant

* *p* < .05

** *p* < .01

*** *p* < .001.

The ability of a BFG sample to absorb water is an essential property during its reconstitution in hot water to eba and is a function of smaller granule sizes and, thus, higher solubility (Awoyale et al., 2020). The WAC of the BFG ranged from 520.00% to 627.14%. This implies that BFG produced from the NR292D variety (627.14%) will absorb more water during its preparation to eba than BFG produced from the TMEB419 variety (520.55%) with lower WAC (Table 1). The WAC of this study agreed with the WAC of the spontaneously fermented gari produced from different cassava varieties (140.64%–693.18%) (Awoyale et al., 2020). However, the WAC of the commercially available spontaneously fermented gari in Nigerian markets (450.46%–514.70%) was lower than that of the present study (Awoyale et al., 2017). Conversely, the WAC (423.13%–519.89%) of the BFG reported by Awoyale et al. (2021a) was lower than the present study. These variations in WAC may be attributed to different mixing ratios of the fresh and the backslopped cassava mash.

A good quality gari could swell at least three times its original volume (Awoyale et al., 2020). Gari produced from NR130022 (16.19%) had the highest SWP compared with gari made from TMEB419 (8.31%) with lower SWP (Table 1). All the BFG may be of good quality since there was no significant difference (*p* > .05) in their SWP. The SWP (8.23%–12.74%) of the spontaneously fermented gari produced from different varieties reported by Awoyale et al. (2020) falls within the range of values of SWP of the BFG in the present study. On the contrary, the SWP of the BFG produced from different blend ratios of fresh and backslopped fermented cassava mash (22.45%–30.95%) was higher than the SWP of the present study (Awoyale et al., 2021a). Differences in blend ratios of the fresh and backslopped cassava mash may be responsible for the variations in SWP.

The degree of leaching of the straight‐chain component of starch (amylose) from the granules during swelling is related to the SI and is affected by intermolecular forces and the presence of surfactants and other associated substances (Moorthy, 2002). The SI of the BFG ranged between 9.76% and 23.00% (Table 1). This means that the BFG produced from TMEB419 may consist of very much associated starch granules with a large and highly bonded micellar structure because of its lower SI, while the BFG from IITA‐TMSIBA000070 may be made up of weak associated forces owing to its high SI (Singh et al., 2004). The SI of commercially available spontaneously fermented gari in Nigerian markets (3.06%–4.18%) (Awoyale et al., 2017) and spontaneously fermented gari produced from different varieties (2.18%– 8.23%) (Awoyale et al., 2020) was lower than that of this study.

Ikujenlola (2008) observed that any product’s BD is essential in choosing the suitable packaging material. This is because the higher the amount of the product that could be packaged in each container volume, the lower the BD value, and thus, the lower the packaging and transportation costs. The BD of the BFG was higher in the NR292D variety (66.68%) and lower in TMSF1153P0001 (49.42%) (Table 1), which means that more of the BFG produced from TMSF1153P0001 may be packaged in a smaller space compared to that of NR292D because of its lower BD. The BD (40%–70%) reported by Awoyale et al. (2020) for gari produced from different cassava varieties was like that of the present study, although the BD of the BFG made from NR292D was higher than the BD values (43.85%–56.84%) reported by Awoyale et al. (2017) for commercially available spontaneously fermented gari in Nigerian markets. Also, the values reported for the BD (55.25%–64.11%) of the BFG produced from different ratios of fresh and backslopped cassava mash were within the range of values reported in this study (Awoyale et al., 2021a).

The dispersibility of any starchy food is a measure of its reconstitutability in water. There is a positive correlation between the dispersibility of starchy foods and their reconstitution in water (Kulkarni & Ingle, 1991). The BFG produced from NR292D (43.50%) had the highest dispersibility, and that from TMS13F1343P0044 (22%) had the lowest (Table 1). This implied that the NR292D BFG might reconstitute appropriately in hot water without lumps during the preparation of eba because of its high dispersibility compared with the BFG produced from TMS13F1343P0044 with lower dispersibility. However, lump formation may be affected by how the gari sample was added to the boiled water during reconstitution (Awoyale et al., 2020). The dispersibility of the BFG of the present study was within the range of values (20.25%–75.25%) of the spontaneously fermented gari produced from different cassava varieties (Awoyale et al., 2020), as well as the range of values (53.50%–66.00%) reported for the BFG produced from different ratios of fresh and backslopped cassava mash (Awoyale et al., 2021a).

### 3.2 **Pasting properties of** BFG **from different cassava varieties**

The pasting properties are essential in predicting the behavior of gari during and after cooking since it may be reconstituted with hot water into eba (Adebowale et al., 2007). The pasting properties of the BFG showed that mean peak viscosity is 407.78 RVU, trough viscosity 234.88 RVU, breakdown viscosity 172.90 RVU, final viscosity 388.74 RVU, setback viscosity 153.86 RVU, peak time 4.86 min, and pasting temperature 78.24°C (Table 2).

**TABLE 2:** Pasting properties of backslopped fermented gari produced from different cassava varieties

Varieties	Peak viscosity (RVU)	Trough viscosity (RVU)	Breakdown viscosity (RVU)	Final viscosity (RVU)	Setback viscosity (RVU)	Peak time (min)	Pasting temp. (°C)
IITA‐TMSIBA000070	396.71 ± 31.29b	214.00 ± 0.24bc	182.71 ± 31.52b	324.50 ± 2.36c	110.50 ± 2.59e	4.77 ± 0.05b	78.33 ± 0.04a
NR292D	395.38 ± 18.67b	207.25 ± 2.94bc	188.13 ± 15.73b	347.34 ± 2.35c	140.08 ± 5.30d	4.67 ± 0.00b	78.78 ± 0.60a
TMEB419	592.46 ± 6.31a	352.67 ± 11.55a	239.80 ± 17.85a	500.67 ± 7.54a	148.00 ± 4.00cd	4.67 ± 0.00b	78.33 ± 0.11a
NR130022	394.29 ± 13.49b	203.63 ± 4.77bc	190.67 ± 8.72b	394.75 ± 6.12b	191.13 ± 1.35a	4.60 ± 0.10b	79.53 ± 0.67a
TMS13F1343P0044	223.25 ± 28.64c	192.84 ± 20.63c	30.42 ± 8.01c	352.67 ± 30.17c	159.83 ± 9.55c	5.57 ± 0.42a	75.38 ± 19.06a
TMSF1153P001	444.63 ± 45.67b	238.92 ± 24.63b	205.71 ± 21.04ab	412.54 ± 22.57b	173.63 ± 2.06b	4.90 ± 0.04b	79.10 ± 0.07a
Mean	407.78	234.88	172.9	388.74	153.86	4.86	78.24
*p* level	***	***	***	***	***	*	NS

Note

Means with different letters within the same column are significantly different (*p* < .05).

Abbreviation: NS, not significant.

* *p* < .05

*** *p* < .001.

The pasting temperature of the BFG samples ranged from 75.38 to 79.53°C, with the BFG produced from the NR130022 variety having the highest and that from TMS13F1343P0044 the lowest, although they were not significantly different (*p* > .05; Table 2). The measure of the smallest temperature expected to cook a given food sample is known as the pasting temperature. This pasting attribute also has implications for other components’ stability and indicates energy costs (Newport Scientific, 1998). Due to the statistical similarities of the pasting temperatures of all the BFG samples, the reconstitution of the BFG produced from all the varieties into eba may consume little energy (Newport Scientific, 1998). The pasting temperature of spontaneously fermented gari (60.14–84.55°C) produced from different varieties reported by Awoyale et al. (2020) agrees with the present study. The BFG samples’ pasting temperature falls within the range of values (69.58–80.40°C) reported for different spontaneously fermented gari samples available in the Nigerian market (Awoyale et al., 2017). In addition, a similar pasting temperature (76.35–79.13°C) was reported for the BFG produced from different ratios of fresh and backslopped cassava mash (Awoyale et al., 2021a). The peak time, which measures the cooking time, is higher in the BFG produced from TMS13F1343P0044 (5.57 min) and lower in that from NR130022 (4.60 min), although all the BFG samples may form a paste in less than six minutes (Adebowale et al., 2007). This finding agreed with Awoyale et al. (2020) on the peak time of spontaneously fermented gari produced from different varieties, as well as the peak time of the BFG made from different ratios of fresh and backslopped cassava mash (Awoyale et al., 2021a).

The peak viscosity of the BFG ranged between 223.25 RVU and 592.46 RVU. The BFG produced from the TMEB419 variety had the highest value, and TMS13F1343P044 had the lowest (Table 2). The maximum viscosity developed during or soon after the heating portion, which contributes to the good texture of starchy paste, is known as the peak viscosity (Ikegwu et al., 2009). Hence, gari consumers that prefer good textured eba may use the BFG produced from TMEB419 because of its high peak viscosity compared to the BFG from TMS13F1343P0044 with low peak viscosity (Ikegwu et al., 2009). It is essential to add that the quantity of water added during the reconstitution of gari into eba affects the texture. The peak viscosity of the BFG produced from the TMS13F1343P0044 variety (223.25 RVU) in the present study was within the range of values (129.17–241.30 RVU) of the spontaneously fermented gari available in the Nigerian markets (Awoyale et al., 2017). The peak viscosity of the BFG produced from all the cassava varieties in the present study except for that of TMS13F1343P0044 falls within the peak viscosity values (371.69–680.99 RVU) of the spontaneously fermented gari (Awoyale et al., 2020). Also, the values reported for the peak viscosity (298.46–419.08 RVU) of the BFG were produced from different ratios of fresh and backslopped cassava mash (Awoyale et al., 2021a) was within the range of values in the present study.

The higher the breakdown viscosity, the lower the ability of the sample to withstand heating and shear stress during cooking (Adebowale et al., 2007). Consequently, the BFG produced from TMEB419 with the highest breakdown viscosity (239.80 RVU) may not withstand heating and shear stress during cooking into eba compared with the BFG from TMS13F1343P0044 (30.42 RVU) with low breakdown viscosity. The breakdown viscosity of the BFG produced from TMS13F1343P0044 falls within the range of values reported for commercially available spontaneously fermented gari in the Nigerian market by Awoyale et al. (2017), although the breakdown viscosity of the BFG produced from all the other varieties in the present study was higher than the values reported by Awoyale et al. (2017) for spontaneously fermented gari. The breakdown viscosity (124.17–218.75 RVU) of the BFG produced from different blend ratios of fresh and backslopped cassava mash was within the range of values of this study (Awoyale et al., 2021a).

The final viscosity is the most used pasting parameter to determine the quality of a starchy food as it indicates the ability of the material to form a gel after cooking (Sanni et al., 2006). The BFG produced from TMEB419 (500.67 RUV), with the highest final viscosity, may form a gel more quickly after cooking than the BFG from IITA‐TMSIBA000070 (324.50 RVU) with the lowest final viscosity (Table 2). The final viscosity of the spontaneously fermented gari (338.46–507.38 RVU) produced from different varieties (Awoyale et al., 2020) agrees with the final viscosity of the BFG in the present study. Conversely, the final viscosity of spontaneously fermented gari (188.70–318.34 RVU) available in the Nigerian market (Awoyale et al., 2017), as well as that of the BFG produced from different blend ratios of fresh and backslopped cassava mash (266.80–321.63 RVU) were lower than that of this study (Awoyale et al., 2021a).

The stage where retrogradation or re‐ordering of starch molecules occurs is known as the setback viscosity. Lower setback viscosity during the paste’s cooling indicates greater resistance to retrogradation (Adebowale et al., 2007; Sanni et al. 2004). This means that eba produced from IITA‐TMSIBA000070 BFG (110.50 RVU) might not weep easily due to its lower setback viscosity, related to eba produced from NR130022 BFG (191.13 RVU) because of its high setback viscosity (Table 2). The setback viscosity of IITA‐TMSIBA000070 BFG falls within the range of values of the setback viscosity reported by Awoyale et al. (2017) and Awoyale et al. (2020) for different spontaneously fermented gari samples. The setback viscosity of the BFG produced from different varieties agrees with the range of values (74.92–177.58 RVU) reported by Awoyale et al. (2020) for spontaneously fermented gari, except for that of NR130022 which was higher. Some of the values reported for the setback viscosity of the BFG produced from different ratios of fresh and backslopped cassava mash fall within the range of values of the present study (Awoyale et al., 2021a).

### 3.3 Chemical composition of BFG from different cassava varieties

The chemical composition of gari produced from different varieties showed that the mean of the starch is 75.15%, sugar 2.83%, amylose 29.63, ash 0.86%, pH 5.00, cyanogenic potential (CNP) 1.12 mg HCN/kg, protein 1.71% and fat 0.59% (Table 3).

**TABLE 3:** Chemical composition of backslopped fermented gari produced from different cassava varieties

Varieties	Starch content (%)	Sugar content (%)	Amylose content (%)	Ash content (%)	pH value	CNP (mg HCN/kg)	Protein content (%)	Fat content (%)
IITA‐TMSIBA000070	76.55 ± 0.14ab	3.43 ± 0.06a	32.40 ± 0.10b	0.83 ± 0.02cd	4.94 ± 0.01c	1.94 ± 0.11a	1.83 ± 0.00b	0.73 ± 0.03a
NR292D	77.10 ± 0.53a	3.59 ± 0.12a	33.62 ± 0.22a	0.96 ± 0.01a	5.21 ± 0.05b	0.53 ± 0.00c	1.65 ± 0.02 cd	0.76 ± 0.01a
TMEB419	71.92 ± 0.03c	2.68 ± 0.11b	27.29 ± 0.05e	0.82 ± 0.01de	4.72 ± 0.02d	0.71 ± 0.14c	1.75 ± 0.00bc	0.27 ± 0.06c
NR130022	75.70 ± 0.79b	2.56 ± 0.04b	28.19 ± 0.32d	0.91 ± 0.03b	4.05 ± 0.01f	1.23 ± 0.14b	1.41 ± 0.04e	0.66 ± 0.04a
TMS13F1343P0044	77.13 ± 0.09a	2.16 ± 0.05c	26.04 ± 0.28f	0.87 ± 0.01c	4.58 ± 0.01e	1.77 ± 0.12a	2.03 ± 0.10a	0.72 ± 0.06a
TMSF1153P001	72.51 ± 0.30c	2.56 ± 0.03b	30.23 ± 0.06c	0.78 ± 0.02e	6.51 ± 0.01a	0.51 ± 0.18c	1.58 ± 0.02d	0.40 ± 0.02b
Mean	75.15	2.83	29.63	0.86	5	1.12	1.71	0.59
*p* level	***	***	***	***	***	***	***	***

Note

Means with different letters within the same column are significantly different (*p* < .05).

*** *p* < .001.

Akingbala et al. (2005) reported that starch content is one of the vital quality indices of gari which determine the texture of eba. The starch content was higher in the BFG produced from TMS13F1343P0044 (77.13%), and lower in the BFG made from TMEB419 (71.92%). The conversion of the starch into sugar during fermentation was more in the BFG from NR292D (3.59%) and lower in that of the TMS13F1343P0044 (2.16%) (Akingbala et al., 2005) (Table 3). The starch content of the gari (82.62%–92.00%) produced from different varieties using the spontaneous fermentation methods was higher than in the BFG in the present study, although the sugar content of the BFG made from NR292D falls within the range of values (2.78%–4.29%) reported for spontaneously fermented gari (Awoyale et al., 2020). This could be attributed to the varieties used and different methods of fermentation. Also, the starch content of the present study falls within the range of values (72.04%–81.86%) reported for the BFG produced from different ratios of fresh and backslopped cassava mash (Awoyale et al., 2021b).

The amylose content of starchy foods determines the stability of the viscous solution formed when starch is heated (Awoyale et al., 2015). Thus, the higher the amylose content of the gari, the higher the retrogradation of the starch molecules after cooking into eba. This implied that eba prepared from NR292D BFG (33.62%) might exhibit a more increased rate of retrogradation because of its high amylose content, compared to the BFG produced from TMS13F1343P0044 (26.04) with low amylose content (Table 3). The amylose content (28.35%–30.24%) of the BFG made from different blend ratios of fresh and backslopped cassava mash (Awoyale et al., 2021b) was within the range of values reported in this study.

The ash content of the BFG ranged between 0.78% and 0.96%, with the BFG produced from NR292D having the highest and that from TMSF1153P001 the lowest (Table 3). Ash content suggests the possible mineral status in a sample, although contamination during processing could show an elevated concentration in a sample (Baah et al., 2009). The BFG produced from different cassava varieties falls below the stipulated value of the Codex Alimentarius Commission of 1.5% (Codex Alimentarius Commission, 1985). However, the ash content of the present study is less compared to the values (1.43%–1.60%) reported for BFG produced from different ratios of fresh and backslopped cassava mash (Awoyale et al., 2021b). This may be attributed to different cassava varieties and blend ratios of the fresh and backslopped cassava mash.

The pH value measures the degree of acidity or alkalinity of fermented products (Sanni et al., 2005). The pH value of the BFG samples was higher fromTMSF1153P001 (6.51) and lowered from NR130022 (4.05) (Table 3). This implied that the BFG produced from NR130022 would be sourer when consumed than the BFG from TMSF1153P001, which may be bland in taste (Onasoga et al., 2014). The breakdown of starch in the fresh cassava roots by *Corynebacterium manihot* to simple sugars and its subsequent fermentation to produce lactic and formic acids may be responsible for the low pH values in some of the gari (Amund & Ogunsina, 1987). The pH values (4.05–6.55) of the spontaneously fermented gari produced from different varieties reported by Awoyale et al. (2020) were within the range of values of the present study.

Cassava contains hydrogen cyanide, which occurs because of the hydrolysis of cyanogenic glucosides, a group of nitriles‐containing compounds that yield cyanide following an enzymatic breakdown. This cyanogenic glycoside is toxic to humans if the cassava root is not adequately processed before consumption (Uyo et al., 2007). The BFG CNP content ranged from 0.51 to 1.94 mgHCN/kg, with the BFG produced from IITA‐TMSIBA000070 having the highest value and the lowest from TMSF1153P001 (Table 3). The CNP content of all the BFG samples is very low compared to the Codex Alimentarius Commission standard of 10 mg HCN/kg (Codex Alimentarius Commission, 1985). The CNP content of the BFG samples in this study was within the range of values reported in a previous study for gari produced from different varieties using spontaneous fermentation methods (Awoyale et al., 2020), although lower than the values (2.14–3.77 mg HCN/kg) reported for BFG made from different ratios of fresh and backslopped cassava mash (Awoyale et al., 2021b).

The protein and fat contents of cassava‐based products are very low (Awoyale et al., 2021b), hence the low protein and fat contents of the BFG. The protein content of the BFG ranged from 1.41% to 2.03%, with the BFG produced from the TMS13F1343P0044 variety having the highest and that of the NR130022 variety the lowest. The protein content of the BFG produced from the TMS13F1343P0044 variety was higher compared to the protein content reported for the BFG (0.86%–1.41%) and spontaneously fermented gari (1.07%–1.49%) by Awoyale et al. (2021b). The fat content of the BFG was higher in the NR292D variety (0.76%) and lower in that of the TMEB419 variety (0.27%). The fat content of the BFG (0.08%–0.18%) and the spontaneously fermented gari (0.07%–0.15%) reported by Awoyale et al. (2021b) were lower compared to that of the present study. The variations in protein and fat contents of the BFG may be attributed to the differences in cassava varieties.

### 3.4 Sensory texture profile of eba prepared from different cassava varieties

The mean of the sensory texture profile revealed that the samples of eba prepared from the BFG produced from different varieties were moderately moldable, stretchable, sticky, and soft (Table 4). All the sensory texture attributes of the eba were significantly different (*p* < .05) except for the stretchability, which did not differ significantly (*p* > .05) (Table 4). Ndjouenkeu et al. (2021) reported that a good eba is characterized as smooth, firm, less sticky, elastic, moldable and as having good swelling of the gari during preparation. Also, textural properties of eba such as cohesiveness, mouldability, stretchability, and softness are essential and desired at various levels depending on the region, culture, and personal preferences (Adinsi et al., 2019; Ndjouenkeu et al., 2021).

**TABLE 4:** Sensory texture profile of eba produced from different cassava varieties

Samples	*N*	Mouldability	Stretchability	Stickiness	Hardness
IITA‐TMSIBA000070	28	2.18 ± 0.67ab	1.68 ± 0.61a	2.54 ± 0.51ab	1.75 ± 0.59cd
NR292D	28	1.93 ± 0.86b	1.61 ± 0.63a	2.64 ± 0.56a	1.54 ± 0.51d
TMEB419	28	2.50 ± 0.58a	1.68 ± 0.72a	2.14 ± 0.80c	2.46 ± 0.64a
NR130022	28	2.46 ± 0.51a	1.96 ± 0.64a	2.14 ± 0.59c	2.00 ± 0.61bc
TMS13F1343P0044	28	2.39 ± 0.63a	1.68 ± 0.55a	2.11 ± 0.57c	2.25 ± 0.52ab
TMSF1153P001	28	2.29 ± 0.66ab	1.75 ± 0.65a	2.29 ± 0.71bc	1.93 ± 0.60bc
Mean		2.29	1.73	2.31	1.99
*p* level		*	NS	**	***

Note

Mouldability: 1 = Not mouldable, 2 = Moderately mouldable, 3 = Mouldable. Stretchability: 1 = Not Stretchable, 2 = Moderately stretchable, 3 = Stretchable. Stickiness: 1 = Not Sticky, 2 = Moderately sticky, 3 = Sticky. Hardness: 1 = Hard, 2 = Moderately hard, 3 = Harder.

Abbreviation: NS, not significant.

* *p* < .05

** *p* < .01

*** *p* < .001.

Means with different letters within the same column are significantly different (p<0.05).

The eba from the BFG produced from TMEB419 was more moldable than the eba prepared from the other BFG, which were moderately moldable (Table 4). This means that eba from TMEB419 BFG may be preferred more than the others (Ndjouenkeu et al., 2021). A significant difference (*p* < .05) exists between the mouldability of the eba produced from TMEB419 BFG and NR292D BFG. The mouldability of the eba prepared from different varieties of gari had a significant correlation (*p* < .05) with the functional and pasting properties of the BFG samples, but a significant and negative correlation exists between the mouldability of the eba and the contents of sugar (*p* < .05, *r* = −0.82) and amylose (*p* < .05, *r* = −0.90) of the BFG (Table 5). This implied that the lower the amount of the sugar and amylose content in the BFG, the more moldable the eba prepared from such gari. Also, the higher mouldability of the eba prepared from TMEB419 BFG might be attributed to its lower contents of sugar and amylose. The protein content of the BFG has a negative but not significant correlation with the mouldability (*p* > .05, *r* = −0.23) of the eba (Table 5).

**TABLE 5:** Correlation of the functional and pasting properties and the chemical composition of backslopped fermented gari with the sensory and instrumental texture profile of the eba produced from different varieties

Parameters	Sensory texture attributes				Instrumental texture attributes				
	Moldability	Stretchability	Stickiness	Hardness	Hardness	Adhesiveness	Mouldability	Stretchability	Gumminess
Water absorption capacity	−0.33	0.33	−0.65	0.62	0.08	−0.47	−0.00	−0.25	0.08
Swelling power	−0.45	0.18	−0.28	0.58	0.67	−0.72	−0.46	−0.17	0.68
Solubility index	−0.49	−0.15	−0.11	0.21	0.86*	−0.50	−0.69	−0.01	0.86*
Bulk density	−0.75	−0.83*	−0.46	−0.64	0.14	0.33	−0.08	0.58	0.15
Dispersibility	−0.75	−0.62	−0.81	−0.38	0.27	0.35	−0.21	0.34	0.28
Peak viscosity	0.24	−0.17	−0.07	−0.42	0.08	0.69	−0.28	−0.11	0.07
Trough viscosity	0.42	−0.20	0.25	−0.57	−0.30	0.73	0.05	0.02	−0.31
Breakdown viscosity	0.05	−0.11	−0.31	−0.21	0.37	0.52	−0.50	−0.19	0.36
Final viscosity	0.71	0.22	0.32	−0.21	−0.37	0.52	0.05	−0.31	−0.39
Setback viscosity	0.71	0.92**	0.21	0.75	−0.20	−0.37	0.01	−0.77	−0.22
Peak time	0.07	0.02	0.40	−0.02	−0.54	−0.21	0.69	0.34	−0.53
Pasting temperature	−0.00	0.17	−0.44	0.21	0.57	0.15	−0.64	−0.41	0.56
Sugar content	−0.83*	−0.67	−0.73	−0.37	0.45	0.24	−0.31	0.41	0.46
Starch content	−0.57	−0.09	−0.22	0.29	0.20	−0.67	−0.04	0.13	0.20
Amylose content	−0.86*	−0.57	−0.84*	−0.24	0.46	0.23	−0.22	0.42	0.49
Ash content	−0.36	0.13	−0.46	0.40	−0.01	−0.52	−0.08	−0.23	−0.03
pH value	−0.29	−0.35	−0.47	−0.38	−0.13	0.64	0.40	0.51	−0.10
Protein content	−0.23	−0.53	0.34	−0.55	−0.43	0.12	0.57	0.68	−0.41
Fat content	−0.60	−0.06	−0.28	0.36	0.31	−0.69	−0.12	0.10	0.32

Abbreviation: CNP, cyanogenic acid potential.

* *p* < .05

** *p* < .01.

The eba prepared from NR130022 BFG was more stretchable than that prepared from the other varieties. But there was no significant difference (*p* > .05) in the stretchability of eba prepared from all the varieties BFG, as all were moderately stretchable (Table 4). This implies that all the BFG may be preferred when prepared into eba, as stretchability is one of the essential textural attributes liked by the consumers of eba (Ndjouenkeu et al., 2021). The stretchability of eba had no significant correlation (*p* > .05) with all quality attributes of the BFG samples (Table 5). The protein content of the BFG has a negative but not significant correlation with the stretchability (*p* > .05, *r* = −0.53) of the eba (Table 5).

The samples of eba prepared from IITA‐TMSIBA000070 and NR292D BFG were stickier than those prepared from the other varieties (Table 4). It is imperative to add that the less sticky the eba, the more the acceptability (Adinsi et al., 2019; Ndjouenkeu et al., 2021). Hence, the eba prepared from these varieties may not be acceptable to the consumers based on their stickiness. The stickiness of the eba was positively correlated with dispersibility of the BFG (*p* < .05, *r* = 0.88), as well as the sugar (*p* < .01, *r* = 0.94) and amylose (*p* < .01, *r* = 0.97) contents (Table 5). This means that the higher the dispersibility, and the sugar and amylose content of the BFG, the higher the stickiness of the eba prepared from such gari. Also, the higher stickiness of the eba prepared from the IITA‐TMSIBA000070 and NR292D BFG may be due to higher dispersibility and the contents of sugar and amylose. A positive but not significant correlation exist between the protein content of the BFG and the stickiness (*p* > .05, *r* = 0.34) of the eba (Table 5).

Even though the hardness of the eba prepared from TMEB419 BFG was better than the eba prepared from the other varieties BFG, there was no significant difference (*p* > .05) in the hardness of the eba prepared from TMEB419 BFG and that of TMS13F1343P0044 BFG (Table 4). The hardness of the eba prepared from the different varieties falls within the moderately hard range (Table 4). This means that the eba prepared from all the varieties of the BFG may be acceptable because of their moderately hard texture (Ndjouenkeu et al., 2021). Of all the chemical compositions, it was only the amylose content of the BFG that had a significant but negative correlation (*p* < .01, *r* = −0.92) with the hardness of the eba (Table 5). This means that, the lower the amylose content of the BFG, the harder the eba prepared from such gari. Hence, the hardness of the eba prepared from TMEB419 BFG may be attributed to its low amylose content. The protein content of the BFG has a negative but not significant correlation with the hardness (*p* > .05, *r*= −0.55) of the eba (Table 5).

### 3.5 Instrumental texture profile of eba produced from different cassava varieties

Table 5 shows the instrumental texture profile of the eba prepared from different varieties. The mean of the instrumental texture profiling is hardness 26.91 N/m^2^, adhesiveness −59.83 N/m^2^, mouldability 0.90, stretchability 0.98, and gumminess 23.95 N/m^2^. Significant differences (*p* < .05) exist in all the instrumental texture attributes of the eba, except the mouldability and stretchability, which were not significantly different (*p* > .05) (Table 6).

**TABLE 6:** Instrumental texture profiling of eba produced from different cassava varieties

Samples	Hardness (N/m^2^)	Adhesiveness (N/m^2^)	Mouldability	Stretchability	Gumminess (N/m^2^)
IITA‐TMS‐IBA000070	29.12 ± 3.87ab	−68.56 ± 5.33b	0.84 ± 0.08b	0.96 ± 0.06a	24.15 ± 2.06bc
NR292D	24.41 ± 3.83c	−42.44 ± 11.27a	0.92 ± 0.03ab	0.97 ± 0.16a	22.38 ± 0.07cd
TMEB419	30.12 ± 1.64a	−71.20 ± 3.81b	0.86 ± 0.04b	1.03 ± 0.24a	25.68 ± 1.62ab
NR130022	26.60 ± 1.47bc	−48.09 ± 3.44a	0.90 ± 0.02ab	1.00 ± 0.03a	23.82 ± 1.36bc
TMS13F1343P0044	30.22 ± 0.11a	−64.78 ± 10.34b	0.90 ± 0.01ab	0.88 ± 0.15a	27.10 ± 0.40a
TMS13F1153P001	21.03 ± 1.63d	−63.89 ± 5.10b	0.98 ± 0.14a	1.06 ± 0.03a	20.54 ± 1.38d
Mean	26.91	−59.83	0.9	0.98	23.95
p level	***	***	NS	NS	***

Abbreviation: NS, not significant.

*** *p* < .001.

Means with different letters within the same column are significantly different (p<0.05).

Hardness is defined as an indicator of the most direct response to taste, directly affecting chewiness, gumminess, and cohesiveness in the texture profile analysis (Awoyale, et al., 2021; Goddard et al., 2015). The hardness of the eba prepared from TMS13F1343P0044 BFG (30.22 N/m^2^) was significantly (*p* < .05) more than that of the eba prepared from TMSF1153P001 BFG (21.03 N/m^2^) (Table 6). The hardness of the eba from the TMS13F1343P0044, TMEB419, and IITA‐TMS‐IBA000070 BFG was not significantly different (*p* > .05). The hardness of the eba has no significant correlation (*p* > .05) with all quality attributes of the BFG (Table 5).

In the food field, the stickiness can be defined as the negative force generated when a food sample is subjected to pressure deformation (Goddard et al., 2015). Stickiness is also related to adhesiveness. Adhesiveness is the degree to which the eba sticks to the hand, mouth surface, or teeth (Awoyale, et al., 2021). The adhesiveness of the eba ranged from −71.20 to −42.44 N/m^2^, with the eba prepared from the NR292D BFG having the highest value (*p* < .05) and the eba prepared from the TMEB419 BFG having the lowest value. The adhesiveness of the eba prepared from the NR292D BFG was not significantly different (*p* > .05) from that of the NR130022 BFG (Table 6). The adhesiveness of the eba was significant and negatively correlated with the water absorption capacity (*p* < .05, *r* = −0.93), solubility index (*p* < .05, *r* = −0.89) and the ash content (*p* < .05, *r* = −0.87) of the BFG (Table 5). This means that the lower the water absorption capacity, solubility index, and ash content of the BFG, the higher the adhesiveness of the eba prepared from such gari. Hence, the low adhesiveness of the eba prepared from the TMEB419 BFG may be attributed to its high ash content.

Cohesiveness/mouldability is how well the product withstands a second deformation relative to its resistance under the first deformation. It is calculated as the work area during the second compression divided by the work area during the first compression (Goddard et al., 2015). Usually, the eba is squeezed manually, during which the mechanical and geometrical characteristics are assessed, molded into balls with the hand, dipped into the soup, and then swallowed. The mouldability of the eba ranged from 0.84 in the IITA‐TMS‐IBA000070 BFG to 0.98 in the TMS13F1153P001 BFG (Table 6). There were no significant differences in the mouldability of the eba prepared from all the varieties. The mouldability of the eba has no significant correlation (*p* > .05) with all the quality attributes of the BFG (Table 5).

Stretchability or elasticity is the degree to which the eba returns to its original shape after compression between the teeth (Awoyale, et al., 2021; Goddard et al., 2015). The stretchability was lower in the eba prepared from the TMS13F1343P0044 BFG (0.88) and higher in the eba prepared from the TMS13F1153P001 BFG (1.06) (Table 6). The correlation between the stretchability of the eba and the peak viscosity (*p* < .05, *r* = 0.83), breakdown viscosity (*p* < .05, *r* = 0.88), and the pasting temperature (*p* < .05, *r* = 0.82) of the BFG was significant and positive, while a significant negative correlation (*p* < .05, *r* = −0.83) exist between the stretchability of the eba and the starch content of the BFG (Table 5). This means that the higher the peak and breakdown viscosities of the BFG, the higher the stretchability of the eba prepared from such gari. The high peak and breakdown viscosity of the BFG prepared from the TMS13F1153P001 variety may be responsible for the high stretchability of its eba.

The energy required to disintegrate a semi‐solid food until it can be swallowed is known as gumminess. It is calculated as cohesiveness multiplied by the hardness (Awoyale, Alamu, et al., 2021; Goddard et al., 2015). The gumminess of the eba prepared from the TMS13F1343P0044 BFG (27.10 N/m^2^) was significantly (*p* < .05) higher than that of the TMS13F1153P001 BFG (20.54 N/m^2^) (Table 6). Although the gumminess of the eba prepared from the TMS13F1343P0044 BFG was not significantly different (*p* >.05) from that of the eba prepared from the TMEB419 BFG (Table 6). This could be attributed to differences in the varieties used. The hardness, mouldability, and gumminess of the eba have no significant correlation (*p* > .05) with all the functional and pasting properties, and the chemical composition of the BFG (Table 5).

## 4 CONCLUSIONS

A significant and negative correlation exists between the STPA mouldability of the eba and the sugar and amylose contents of the BFG. The STPA stretchability of the eba had a significant negative correlation with the bulk density and a significant positive correlation with the setback viscosity of the BFG. A significant and negative correlation exists between the STPA stickiness of the eba and the amylose contents of the BFG. The ITPA adhesiveness of the eba was significant and negatively correlated with the water absorption capacity, solubility index, and ash content. The correlation between the ITPA stretchability of the eba and the peak viscosity, breakdown viscosity, and the pasting temperature of the BFG was significant and positive, while a significant negative correlation exists between the ITPA stretchability of the eba and the starch content of the BFG. The information provided in this study to breeders may help breed cassava varieties that will be acceptable to end‐users in terms of the quality characteristics of gari and the textural attributes of the eba.

## CONFLICT OF INTEREST

The author declares that there is no conflict of interest that could be perceived as prejudicing the impartiality of the research reported.

## AUTHOR CONTRIBUTIONS

**Wasiu Awoyale:** Conceptualization; Formal analysis; Investigation; Methodology; Visualization; Writing‐original draft; Writing‐review & editing. **Hakeem Abolore Oyedele:** Conceptualization; Formal analysis; Investigation; Methodology; Visualization; Writing‐original draft; Writing‐review & editing. **Ayodele A Adenitan:** Conceptualization; Formal analysis; Investigation; Methodology; Visualization; Writing‐original draft; Writing‐review & editing. **Michael Adesokan:** Project administration; Supervision; Writing‐review & editing. **Oladeji Emmanuel Alamu:** Project administration; Supervision; Writing‐review & editing. **Busie Maziya‐Dixon:** Conceptualization; Investigation; Methodology; Project administration; Supervision; Visualization; Writing‐original draft; Writing‐review & editing.

## ETHICAL APPROVAL

The authors of this study declare that the sensory evaluation followed the tenets of the Declaration of Helsinki promulgated in1964 and was approved by the institutional ethical review committee. In addition, verbal consent was obtained from the participants.

## Data Availability

The data that support the findings of this study are available from the corresponding author upon reasonable request.
